# Development of Local interventions within JADECARE – Key learnings from two Next Adopters

**DOI:** 10.1093/eurpub/ckac129.509

**Published:** 2022-10-25

**Authors:** R Papa, Z Dolecek

**Affiliations:** 1 Regional Health Agency Marche Region, Ancona, Italy; 2 University Hospital Olomouc, Olomouc, Czechia

## Abstract

In this third presentation, the NA representatives Roberta Papa (Regional Health Agency Marche Region, Italy) and Zdislav Dolecek (University Hospital Olomouc, Czech Republic), will present the Local interventions and action plans they developed during the pre-implementation phase, pre-final results of their implementation phase and preliminary lessons learned from the implementation.

